# 25 Hydroxyvitamin D Serum Concentration and COVID-19 Severity and Outcome—A Retrospective Survey in a Romanian Hospital

**DOI:** 10.3390/nu15051227

**Published:** 2023-02-28

**Authors:** Adriana Topan, Mihaela Lupse, Mihai Calin, Cristian Jianu, Daniel-Corneliu Leucuta, Violeta Briciu

**Affiliations:** 1 Department of Infectious Diseases, “Iuliu Hatieganu” University of Medicine and Pharmacy, 400348 Cluj-Napoca, Romania; 2 The Clinical Hospital of Infectious Diseases, 400348 Cluj-Napoca, Romania; 3 Department of Medical Informatics and Biostatistics, “Iuliu Hatieganu” University of Medicine and Pharmacy, 400349 Cluj-Napoca, Romania

**Keywords:** 25(OH)D deficiency, vitamin D deficiency, COVID-19, severity form, ICU need, mortality, comorbidities

## Abstract

Interest in the immunomodulatory function of vitamin D has grown since the COVID-19 pandemic started. Our study investigated the possible association between vitamin D deficiency and COVID-19 severity, intensive care needs, and mortality in patients hospitalized with COVID-19. A prospective cohort study was performed on 2342 COVID-19 hospitalized patients between April 2020 and May 2022 in a Romanian tertiary hospital for infectious diseases. A multivariate generalized linear model for binary data was fit with dependent variables: severe/critical form of COVID-19, intensive care need, and fatal outcome as a function of vitamin D deficiency, controlling for age, comorbidities, and vaccination status. More than half of the patients (50.9%) were classified with vitamin D deficiency based on a serum concentration of less than 20 ng/mL. There was a negative association between vitamin D and age. Vitamin D-deficient patients presented with more cardiovascular, neurological, and pulmonary diseases, as well as diabetes, and cancer. In multivariate logistic regression models, vitamin D-deficient patients had higher odds of severe/critical forms of COVID-19 [OR = 1.23 (95% CI 1.03–1.47), *p* = 0.023] and higher odds of death [OR = 1.49 (95% CI 1.06–2.08), *p* = 0.02]. Vitamin D deficiency was associated with disease severity and death outcome in hospitalized COVID-19 patients.

## 1. Introduction

COVID-19 therapeutics is challenging due to the lack of ideal treatments and new SARS-CoV-2 variants [1,2]. The most recent WHO guidelines on COVID-19 therapeutics, based on a total of 5398 trials, and registered at the beginning of January 2023, show that there are still some uncertainties regarding the impact of treatment on patient outcomes [3]. Vaccinations significantly reduce hospitalizations and mortality in COVID-19 but their effectiveness may vary in time, depending on booster doses [4,5]. Associated risk factors for severe outcomes have been documented, such as old age and comorbidities (e.g., cardiovascular diseases, cancer, diabetes, obesity, and chronic kidney disease) [6,7,8].

Despite vaccination prophylaxis and treatment recommendations, global COVID-19 mortality was still 1.02% at the beginning of February 2023 [9]. In view of these data, COVID-19 still requires more potent therapy.

Since the beginning of the COVID-19 pandemic, there has been increased research on vitamin D’s potential to lower the risk of COVID-19 severity. Vitamin D is essential for bone health and has significant non-skeletal benefits [10]. Vitamin D is a steroid prohormone whose synthesis begins at the skin’s surface, where the 7 dehydrocholesterol precursor is converted into cholecalciferol by the sunlight’s ultraviolet B rays. The diet only provides a small amount of vitamin D. After the first hydrolyzation step in the liver, 25 hydroxyvitamin D (25(OH) D) or calcifediol is produced, which is the major circulating form of vitamin D utilized in clinical settings to determine the body’s vitamin D status. The second hydrolyzation process of vitamin D occurs at the kidney level, producing 1,25 dihydroxy vitamin D (1,25(OH)_2_ vitamin D) or calcitriol, the active metabolite, with its main functions in intestinal calcium, phosphorus absorption, bone mineralization, as well as in immune and tumoral cell differentiation [11,12].

In response to the European Commission’s request, the European Food Safety Authority’s dietary reference vitamin D value is 20 ng/mL. This represents an appropriate target value obtained from cutaneous synthesis and dietary intake and can be used as a marker of vitamin D status in both adult and pediatric populations [13].

Calcitriol is a steroid hormone that interacts with specific vitamin D receptors (VDRs). VDRs are found in a variety of extraskeletal tissues [14,15], including immune system cells, such as activating CD4, CD8 T cells, neutrophils, and antigen-presenting cells. Moreover, 1,25(OH)_2_ vitamin D induces an innate antiviral mechanism in response to viral infections, such as rhinovirus, respiratory syncytial, and influenza virus [16]. Experimental studies have shown that it induces upregulations in cathelicidin and beta-defensins, both of which have important antimicrobial roles [17,18]. Vitamin D intervenes in adaptative immunity by suppressing Th1 cytokines and, subsequently, the production of cytotoxic T cells, decreasing Th17, reducing inflammation, and promoting regulatory T cells. The main result is a decrease in the cytokine storm that leads to serious viral infections, such as COVID-19 [18], as uncontrolled inflammation is the main cause of COVID-19 severity [19,20].

In view of these data, the main objective of our study was to establish a possible association between vitamin D deficiency in patients hospitalized with COVID-19 and COVID-19 severity, intensive care needs, and in-hospital mortality. Our secondary objective was to characterize vitamin D-deficient patients regarding age and associated diseases.

## 2. Materials and Methods

### 2.1. Study Design and Setting

We conducted a retrospective study at the Clinical Hospital of Infectious Diseases Cluj-Napoca, a tertiary infectious disease hospital in Romania. Beginning in March 2020, the hospital was transformed into a first-line hospital for COVID-19 patients.

### 2.2. Participants

Inclusion criteria: diagnosis of COVID-19 (based on a positive SARS-CoV-2 rapid antigen test or a SARS-CoV-2 molecular test), hospitalization between 1 March 2020 and 31 May 2022, age ≥ 18 years old, with a serum concentration of 25(OH)D (assessed on admission day).

Exclusion criteria: 25(OH)D not performed on admission day.

### 2.3. Vitamin D Analysis

Measurement of serum 25(OH)D was performed by chemiluminescence immunoassay UNICEL Dxl 800, using a fully automatic analyzer Beckman Coulter. Interpretations of 25(OH)D concentrations (according to the reagent kit protocol) were as follows: deficient if <20 ng/mL, insufficient if between 20 and 30 ng/mL, and sufficient if ≥ with 30 ng/mL Similar levels are recommended by the American Endocrine Society [12].

### 2.4. Variables

The data registered were age, sex, and comorbidities (cardiovascular, pulmonary, rheumatological, neurological, renal, hepatic, diabetes mellitus, obesity, and cancer). Vaccination status was recorded as unvaccinated, incomplete primary vaccination (meaning one out of two doses if the primary series had two doses), complete primary vaccination, and the booster dose. The value of the serum concentration of 25(OH)D (upon admission) was registered.

COVID-19 severity classification was as follows: asymptomatic, mild (without pulmonary involvement), medium (pulmonary involvement but with oxygen saturation > 93% on room air), severe (severe: more than 30 breaths/min or oxygen saturation < 93% at rest or PaO2/FIO2 < 300 mmHg, and critical: respiratory failure requiring mechanical ventilation, shock, and/or other organ failures that need intensive care), according to the WHO classifications [21]. The severity of COVID-19 was evaluated at discharge. Based on the diagnosis found in the electronic health records of patients and using the 10th revision of the International Statistical Classification of Diseases [22], infectious disease specialists classified the comorbidities into the previously mentioned groups.

Intensive care unit (ICU) stays and in-hospital mortality were recorded.

The hospital’s ethics committee approved the study. Informed consent was obtained from each patient upon admittance.

### 2.5. Statistical Analyses

Quantitative data that were not normally distributed are shown as median and interquartile ranges. The Chi-squared or Fisher’s exact tests were used to compare categorical data between two independent groups (in cases of low expected frequencies). We compared non-normally distributed quantitative data between two separate groups using the Wilcoxon rank sum test; for multiple independent groups, we used the Kruskal–Wallis test. Using the cut-off of 20 ng/mL for 25(OH)D, patients were categorized into two groups: <20 ng/mL and >20 ng/mL. A second analysis was conducted on a three-group classification: <20 ng/mL (meaning deficiency), between 20–30 ng/mL (meaning insufficiency), and ≥30 ng/mL (meaning sufficiency). The multivariate logistic regression analysis (adjusted for age ≥ 65, diabetes, obesity, cancer, and cardiac, pulmonary, hepatic, rheumatologic, neurologic diseases, COVID-19 vaccination, and the number of doses) was conducted to evaluate the association between concentrations of 25(OH)D groups, COVID-19 severity, ICU need, and mortality. Furthermore, we verified the association between the 25(OH)D concentration as a continuous variable and the dependent variables adjusted for the same confounders within a multiple logistic regression model, with a smoothing spline for vitamin D concentration, fitted within a general additive model.

*p*-values of less than 0.05 were regarded as statistically significant for all statistical analyses. The statistical studies were performed using R version 4.1.2 [23].

## 3. Results

The study consisted of 2342 patients who fulfilled all inclusion criteria and were hospitalized between 27 April 2020 and 31 May 2022. A total of 1194 (50.9%) patients were classified with vitamin D deficiency based on having less than 20 ng/mL of 25(OH)D.

### 3.1. Patients Characteristics

The demographics and clinical data (according to the 25(OH)D serum concentrations) of patients are presented in Table 1 and Table S1.

More than half of the COVID-19 hospitalized patients were older than 65 years. A high percentage of patients had associated cardiovascular diseases. Obesity was present in almost one-third of the total included patients. A high percentage of our study group presented with severe/critical forms of COVID-19 and 7.64% died during hospitalization. Complete vaccination was recorded in 14.1% of our study group; 4.4% of patients received a booster dose (Table 1).

Vitamin D deficiency was more prevalent in older patients, with more cardiovascular, neurological, and pulmonary diseases, as well as diabetes and cancer. Severe/critical forms of COVID-19, need for intensive care, and death were more prevalent in patients with vitamin D deficiency (*p* < 0.05).

There were no significant differences regarding associated obesity, hepatic, and rheumatological diseases.

### 3.2. 25(OH)D and Age

**Figure 1:**
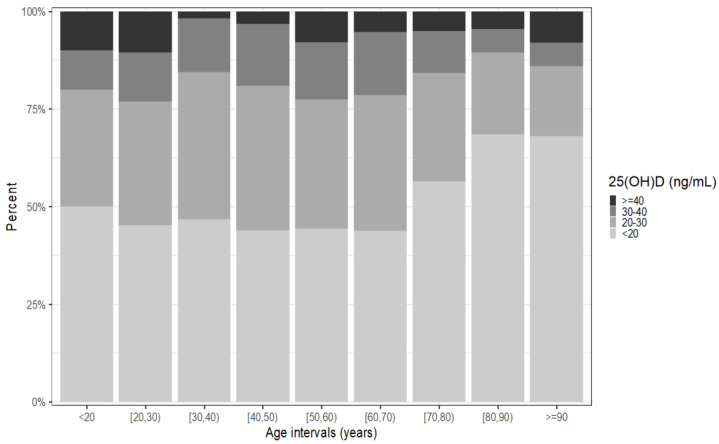
The 25(OH)D serum concentration distribution according to age intervals.

We analyzed the distribution of 25(OH)D serum concentration according to age intervals; the results are presented in Figure 1.

There was an inversely proportional relationship between age and 25(OH)D, i.e., the patient’s age increases as the concentration of vitamin D decreases (*p* < 0.001).

### 3.3. 25(OH)D According to COVID-19 Severity and Death Outcome

**Figure 2:**
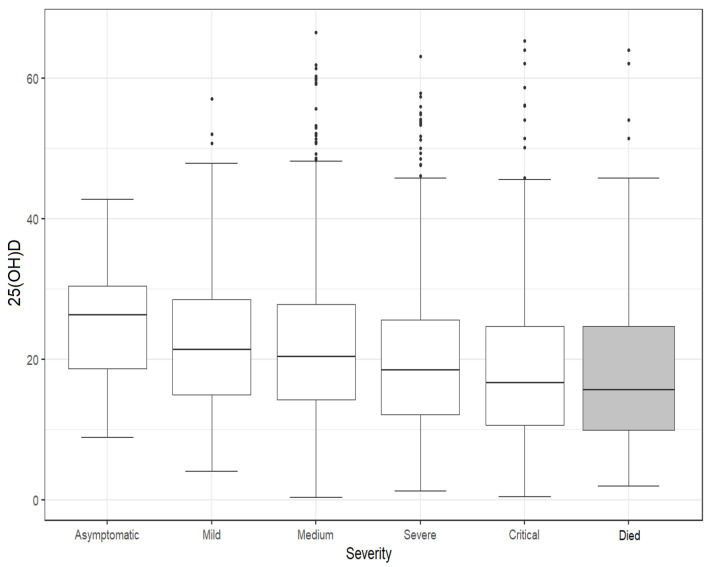
The 25(OH)D serum concentration according to COVID-19 severity and death outcome. The patients who died, with different severity forms, are presented in the grey boxplot. The box shows the median value (horizontal rule), along with the 1st and 3rd quartiles of the observed data (top and bottom of box). Each whisker’s length corresponds to values that are up to 1.5 times the range between the quartiles.

We analyzed the differences in 25(OH)D concentrations according to COVID-19 severity and poor outcomes (death); the results are presented in Figure 2 and Figure S2.

The 25(OH)D concentrations were associated with severity (*p* < 0.001), as higher COVID-19 severity forms are associated with lower vitamin D levels. Patients who died had vitamin D concentrations of [median 15.63 (IQR 9.96–24.73)], 4.42 ng/mL lower (95% CI 2.3–5.36) than survivors (*p* ≤ 0.001).

### 3.4. Multivariate Analyses Predicting Severe/Critical COVID-19, ICU Needs, and Death

Results of the multivariate binary regression models with dependent-variable–severe/critical COVID-19 as a function of 25(OH)D deficiency, controlled for age, associated diseases, and vaccination status are presented in Table 2.

The 25(OH)D < 20 vs. ≥20 ng/mL was significantly associated with a severe/critical form of COVID-19 [OR = 1.23 (95% CI 1.03–1.47), *p* = 0.023]. When considering the three groups of 25(OH)D in the regression model, there was a statistically significant difference between <20 vs. ≥30, which increased the odds of severe/critical forms of COVID-19 (Supplementary Table S2). Furthermore, there was a statistically significant association between the 25(OH)D concentrations as continuous variables and the odds of a severe/critical form of COVID-19 (*p* < 0.001) in a multivariate logistic regression model with the same adjustments. The relationship between 25(OH)D concentrations and the odds of severe/critical forms of COVID-19 was non-linear (Supplementary Figure S1).

The results of the multivariate logistic regression models that predict death as a function of 25(OH)D deficiency, and adjusted for age, comorbidities, and vaccination, are presented in Table 3.

The 25(OH)D < 20 vs. ≥20 ng/mL was significantly associated with evolution to death [OR = 1.49 (95% CI 1.06–2.08), *p* = 0.02]. When considering the three groups of 25(OH)D concentrations in regression, there was a statistically significant difference between <20 and 20–30, which increased the odds of severe/critical forms of COVID-19 (Supplementary Table S3). Furthermore, there was a statistically significant association between the 25(OH)D concentration as a continuous variable and the odds of severe/critical forms of COVID-19 (*p* = 0.039) in a multivariate logistic regression model with the same adjustments. The relationship between 25(OH)D concentrations and the odds of severe/critical forms of COVID-19 was non-linear (Supplementary Figure S1).

The results of the multivariate logistic regression models that predict intensive care need as a function of 25(OH)D deficiency, adjusted for age, comorbidities, and vaccination, are presented in Table 4.

For the models predicting intensive therapy needs, vitamin D status was not statistically significantly associated with the outcome, considering the two (as well as the three) groups of 25(OH)D concentrations (Supplementary Table S4).

## 4. Discussion

The SARS-CoV-2 infection outcomes in individuals are dependent on multiple variables, such as age or different comorbidities, and infection consequences can include asymptomatic hospital admissions, respiratory support requirements, and death. Since the beginning of the COVID-19 pandemic, significant measures to fight this disease were taken, including increasing the supply of personal protective equipment, highlighting the value of social distancing, and authorizing the emergency use of vaccinations and antivirals for therapy.

Although there has been progress in preventing and treating COVID-19, interest in the use of nutraceuticals, particularly vitamin D (as a way to stimulate the immune system and decrease inflammation), has emerged. Numerous observational studies and meta-analyses, investigating the link between low serum 25(OH)D concentration with the prevalence and severity of COVID-19, have been reported [24,25,26,27,28]. According to a meta-analysis (that included 536,105 patients) vitamin D deficiency was not significantly associated with susceptibility to COVID-19 infection or mortality and vitamin D supplements did not improve patients’ prognoses [29]. Another large meta-analysis that involved nearly 2 million adults suggested that vitamin D deficiency/insufficiency increases susceptibility to COVID-19 (and evolving to a severe form), although the risk of bias was high [30]. On the other hand, a recent systematic review and meta-analysis showed that vitamin D supplementation had no effect on the probability of COVID-19 infection but may reduce mortality and prevent ICU admission in COVID-19 patients [31].

A meta-analysis and trial sequential analysis on four randomized clinical trials published in January 2023 suggested an association between vitamin D supplementation and ICU needs for COVID-19 patients [32], although there was a high risk of bias in three out of five trials, while the trial with the lowest risk of bias did not show an association with a shortened length of in-hospital stay.

In this study, we assessed the serum concentrations of 25(OH)Din patients infected with SARS-CoV-2. In our cohort of 2342 COVID-19 patients, we found a high prevalence of vitamin D deficiency (50.9%). Vitamin D deficiency is extremely common worldwide and is considered a global pandemic [12]. In Europe, 40% of people are vitamin D deficiency, according to reports [33]. In the United States and Canada, 24% and 37%, respectively, are vitamin D-deficient [34]. Over one-third of Australia’s population suffers from vitamin D deficiency [35].

According to one study, Romania has a high prevalence of vitamin D deficiency (59%), especially in the elderly and women and during cold seasons [36]. Niculescu et al. (in a study that included 8024 Romanian subjects) reported a higher prevalence of vitamin D deficiency and seasonal variation in older adults [37]. It is important to consider regional variations in 25(OH)D concentrations that are influenced by latitude, genetics, lifestyle, and dietary sources [33,38,39].

More than half of the COVID-19 hospitalized patients were older than 65 years. Patients with vitamin D deficiency were older (median difference 6 years (IQR 4–7)) than non-deficient patients. Low vitamin D blood concentration is linked to osteoporosis, osteomalacia, and sarcopenia; thus, vitamin D deficiency is common in the elderly, especially in those with multiple associated diseases and comedication [40].

A high percentage of patients in our study group had associated cardiovascular comorbidities. We found a significant difference in vitamin D deficiency for the subgroup of patients with cardiovascular diseases. Several studies linked vitamin D deficiency to cardiovascular diseases. The implied mechanisms might be activation of the renin–angiotensin–aldosterone system, abnormal nitric oxide regulation, oxidative stress, or altered inflammatory pathways [41]. A large cross-sectional study from the United States showed that, after correcting for age, sex, ethnicity, and physical activity, there was an inverse relationship between 25(OH)D concentration and blood pressure [42]. According to Wang et al., patients with low concentrations of circulating 25(OH)D were more likely than vitamin D-sufficient controls to experience cardiovascular problems, such as hypertension [43]. In the Framingham heart study, low serum 25(OH)D was linked to a 60% increase in death due to cardiovascular events [44]. On the other hand, in the VINDICATE trial, no benefits on systolic or diastolic blood pressure were observed after high doses of vitamin D supplementation in chronic heart failure patients [45]. Through a variety of direct and indirect processes, vitamin D deficiency may affect cerebrovascular homeostasis and increase the risk and severity of stroke and cognitive dysfunction [46]. In our group of patients, we found a significant difference in vitamin D deficiency for the subgroup of patients with neurological diseases.

Almost one-quarter of our study group patients had associated diabetes. We found a significant difference in vitamin D deficiency for the subgroup of patients with diabetes. The development of insulin resistance and type 2 diabetes may be caused by vitamin D deficiency, as a normal concentration of vitamin D may reduce low-grade inflammation, which is associated with insulin resistance.

Cancer was found in a low percentage of our patients but a significant vitamin D deficiency was found in this subgroup. Vitamin D was suggested to prevent cancer cell proliferation, apoptosis, cell differentiation, angiogenesis, and metastasis [47]. Data show that higher 25(OH)D concentrations inhibit colorectal carcinogenesis, breast cancer, and prostate cancer [48]; however, further research is needed on the benefits of vitamin D supplementation for cancer patients [49].

Previous diagnosed pulmonary diseases were recorded in 12.25% of patients in our study group. A significant difference in vitamin D deficiency was found in the subgroup of patients with associated pulmonary diseases. Numerous studies have found links between low vitamin D levels, affected lung functioning, and increased risk of inflammation, as almost all pulmonary diseases (e.g., acute respiratory distress syndrome, asthma, chronic obstructive pulmonary disease, pneumonia, and tuberculosis, cystic fibrosis) have inflammatory pathogenesis [50]. A meta-analysis of randomized clinical trials on vitamin D regarding acute respiratory infection (performed before the COVID-19 pandemic) showed that vitamin D supplementation reduced the risk of acute respiratory infections by 12% [51].

Obesity was present in almost one-third of the total included patients. A significant difference in vitamin D deficiency was not found in the subgroup of patients with obesity, though obese patients had a median close to the cutoff value of 20 ng/mL [19.85 (IQR 13.82–27.09)]. A systematic review with a meta-analysis of studies that evaluated the association between 25(OH)D concentrations and obesity showed that vitamin D deficiency was associated with obesity, irrespective of age or geographical location [52]. Due to the insufficiently explored effects on vitamin D receptors from adipose tissue, vitamin D deficiency could not be ruled out as a contributing factor to obesity [53].

We did not find significant differences in vitamin D deficiency for the subgroup of patients with rheumatological diseases; this might be explained by the small size of the subgroup (77 patients, 3.29%) and, possibly, by a previous investigation of vitamin D deficiency in this subgroup of patients and supplementary intake for correction. In rheumatology, to prevent glucocorticoid-induced osteoporosis and to lower the risk of fractures, vitamin D supplementation is indicated [54].

We found significant differences in vitamin D sufficiency for the subgroup of patients with endocrine diseases. Low 25(OH)D concentrations have been linked to autoimmune thyroid diseases, such as Hashimoto’s thyroiditis and Graves’ disease [55]. Anti-TPO antibody titers and thyroid volume appear to be associated with vitamin D insufficiency in Hashimoto’s disease, and supplementation was linked to a decrease in antibody titers and TSH levels [56]. A lower percentage of patients with endocrine diseases in our study group presented with vitamin D deficiency, which might also be explained by a previous investigation of vitamin D deficiency in this subgroup of patients and supplementary intake for correction.

The 25(OH)D concentrations were associated with severity (*p* < 0.001), as higher COVID-19 severity forms are associated with lower vitamin D levels. A decrease in the median value of vitamin D was found in our study as the severity form increased, while patients who died had concentrations of vitamin D [median 15.63 (IQR 9.96–24.73)] that were 4.42 ng/mL lower (95% CI 2.3–5.36) than survivors (*p* ≤ 0.001). Bennouar et al. also found lower mean 25(OH)D concentrations in non-survivors (14.1 ± 9.8 ng/mL) compared to survivors (23.9 ± 14.7 ng/mL) [57].

Comorbidities represent an increased risk for COVID-19 severe outcomes in clinical practice. In the statistical analysis, we performed multivariate analyses in order to adjust regression models to the patient’s age, sex, associated diseases, and vaccination status. In the multivariate logistic regression model, we found a statistically significant association between vitamin D deficiency concentration and a severe form of COVID-19 [OR = 1.23 (95% CI 1.03–1.47), *p* = 0.023]. The association remained statistically significant when considering three groups of 25(OH)D concentrations, as well as when using the 25(OH)D as a continuous variable. An interesting finding was the nonlinear relationship between the 25(OH)D concentration and the log odds of a severe/critical form of COVID-19. The odds of a severe form of COVID-19 decreased with higher concentrations and then changed its direction (but remained in the protective zone); however, in the increasing region, the confidence intervals are too wide to be considered strong evidence. Levels of 25(OH)D above 30 ng/mL were found to be protective against severe/critical disease forms in a different trial consisting of 611 patients with COVID-19 and 25(OH)D concentrations assessed at admittance [58].

The multivariate analysis revealed a statistically significant relationship between vitamin D insufficiency concentration levels and death [OR = 1.49 (95% CI 1.06–2.08), *p* = 0.02]. The association remained statistically significant when considering three groups of 25(OH)D concentrations, as well as when using 25(OH)D as a continuous variable. An interesting finding was the nonlinear relationship between the 25(OH)D concentration and the log odds of death, similar to the relationship with the severe form. A high mortality rate was documented in our study group (7.64%). At the end of October 2021, when the Delta variant dominated, Romania held the first position, globally, regarding daily COVID-19 deaths per million persons, while it cumulatively confirmed COVID-19 deaths per million people on 31 May 2022; the end of our study interval was 3307.09 compared to the world level, where it was 774.65 deaths per million people [59]. Similar results were shown by other studies in which 25(OH)D concentrations were measured on the admission day [60,61,62].

While significantly lower 25(OH)D concentrations in moderate and severe COVID-19 diseases were found compared to mild diseases, no correlation was found between the 25(OH)D concentration measured at admittance and inflammatory biomarkers in a Romanian study involving 203 COVID-19 hospitalized patients [63].

Regarding the multivariate logistic regression, predicting intensive therapy needs, 25(OH)D concentrations were not associated with intensive care needs, although recent meta-analyses showed that vitamin D administration resulted in decreased ICU admission in patients with COVID-19 [33,34]. Radujkovic et al. found higher risks of mechanical ventilation needs/deaths in patients with 25(OH)D concentrations lower than 12 ng/mL at admittance [62]. As ICU was frequently overwhelmed, our results could be explained by the inclusion of the multivariate regression model of patients who were transferred to the ICU, but often patients with ICU needs were treated in infectious disease wards by intensivists.

### Limitations and Strength

Our study’s strengths included the large size of the study group and adjustments made for a large number of confounders. Any cause–effect relationship was precluded by the retrospective observational design of our study, implying the possibility of residual confounding. All group differences cannot be accounted for by the multivariate models. The 25(OH)D concentration was assessed on admission day (but irrespective of the day from the onset of the disease). We did not assess pre-analytical factors, such as the fasting versus non-fasting state, or the time of day of the blood sample collection. Vitamin D supplementation prior to admission was not assessed as a part of the study. As COVID-19 standard treatments frequently changed over the two-year pandemic interval, an important limitation of our study is represented by the absence of adjustment in the multivariate analysis to specific COVID-19 medication used during the hospitalization of patients. Another limitation is represented by the absence of adjustment in the multivariate analyses to the wave of the pandemic, as differences in disease severity are associated with different variants of concern of the virus [64].

## 5. Conclusions

More than half of the patients in the study group were classified with vitamin D deficiency based on a serum concentration of less than 20 ng/mL. There was an inversely proportional relationship between age and vitamin D; patients with vitamin D deficiency presented with more cardiovascular, neurological, and pulmonary diseases, as well as diabetes and cancer.

Vitamin D-deficient patients presented with higher percentages of severe/critical forms of COVID-19, and a higher percentage of death (*p* < 0.05). Vitamin D deficiency was associated with disease severity and death outcomes in hospitalized COVID-19 patients in the multivariate analyses.

## Figures and Tables

**Table 1:** Demographics, clinical data, comorbidities, and vaccination status of the study group.

Characteristics	All (n = 2342)	25(OH)D < 20 ng/mL (n = 1194)	25(OH)D ≥ 20 ng/mL (n = 1148)	*p*
Age (years), median (IQR)	65 (50–75)	68 (52–78)	62 (48–72)	<0.001
Age ≥ 65 years (Yes), n (%)	1172 (50.04)	663 (55.53)	509 (44.34)	<0.001
Sex, n (%)				0.075
Female	1282 (54.74)	675 (56.53)	607 (52.87)	
Male	1060 (45.26)	519 (43.47)	541 (47.13)	
Cardiovascular, n (%)	1412 (60.29)	769 (64.41)	643 (56.01)	<0.001
Neurological, n (%)	297 (12.68)	212 (17.76)	85 (7.4)	<0.001
Diabetes, n (%)	509 (21.73)	283 (23.7)	226 (19.69)	0.019
Cancer, n (%)	197 (8.41)	115 (9.63)	82 (7.14)	0.03
Pulmonary, n (%)	287 (12.25)	168 (14.07)	119 (10.37)	0.006
Obesity, n (%)	689 (29.42)	348 (29.15)	341 (29.7)	0.767
Rheumatological, n (%)	77 (3.29)	41 (3.43)	36 (3.14)	0.686
Endocrine, n (%)	176 (7.51)	63 (5.28)	113 (9.84)	<0.001
Hepatic, n (%)	127 (5.42)	66 (5.53)	61 (5.31)	0.819
Renal, n (%)	145 (6.19)	83 (6.95)	62 (5.4)	0.12
ICU stay, n (%)	271 (11.57)	157 (13.15)	114 (9.93)	0.015
Died, n (%)	179 (7.64)	118 (9.88)	61 (5.31)	<0.001
Severe/critical COVID-19, n (%)	992 (42.36)	560 (46.9)	432 (37.63)	<0.001
Vaccinated, n (%)	495 (21.14)	254 (21.27)	241 (20.99)	0.868
Doses, n (%)				0.991
0:	0: 1847 (78.9)	940 (78.73)	907 (79.08)	
1:	1: 61 (2.61)	31 (2.6)	30 (2.62)	
2:	2: 330 (14.1)	171 (14.32)	159 (13.86)	
3:	3: 103 (4.4)	52 (4.36)	51 (4.45)	

IQR, interquartile range; ICU, intensive care unit; 0/1, 0 means unvaccinated, 1 incomplete vaccination; 2, complete primary vaccination; 3, booster dose.

**Table 2:** Multivariate logistic regression with dependent variables and severe/critical forms of COVID-19 adjusted for 25(OH)D status and all other variables.

Characteristics	OR Adjusted	(95% CI)	*p*-Value
Age ≥ 65 years	1.74	(1.42–2.13)	<0.001
Cardiovascular	1.86	(1.52–2.28)	<0.001
Diabetes	1.56	(1.25–1.93)	<0.001
Obesity	1.79	(1.47–2.18)	<0.001
Pulmonary diseases	1.3	(1–1.7)	0.051
Renal diseases	1.74	(1.21–2.52)	0.003
Hepatic diseases	1.11	(0.76–1.63)	0.576
Rheumatic diseases	1.57	(0.97–2.56)	0.068
Neurological diseases	1.84	(1.4–2.42)	<0.001
Cancer	1.32	(0.96–1.81)	0.092
Vaccine Doses (1 vs. 0)	0.53	(0.29–0.93)	0.03
Vaccine Doses (2 vs. 0)	0.54	(0.41–0.7)	<0.001
Vaccine Doses (3 vs. 0)	0.27	(0.16–0.45)	<0.001
Vitamin D (ng/mL) (<20 vs. ≥20)	1.23	(1.03–1.47)	0.023

OR, odds ratio; CI, confidence interval.

**Table 3:** Multivariate logistic regression with the dependent variable (evolution to death) adjusted for 25(OH)D status and all other variables.

Characteristics	OR Adjusted	(95% CI)	*p*-Value
Age ≥ 65 years	2.91	(1.92–4.52)	<0.001
Cardiovascular	2.37	(1.53–3.78)	<0.001
Diabetes	1.12	(0.78–1.59)	0.518
Obesity	1.41	(0.97–2.02)	0.066
Pulmonary diseases	1.18	(0.75–1.8)	0.459
Renal diseases	1.75	(1.03–2.87)	0.03
Hepatic diseases	1.01	(0.46–1.97)	0.975
Rheumatic diseases	0.68	(0.2–1.71)	0.471
Neurological diseases	2.05	(1.38–3)	<0.001
Cancer	1.69	(1–2.75)	0.042
Vaccine Doses (1 vs. 0)	0.79	(0.23–2.05)	0.671
Vaccine Doses (2 vs. 0)	0.53	(0.3–0.89)	0.024
Vaccine Doses (3 vs. 0)	0.59	(0.22–1.3)	0.233
25(OH)D (ng/mL) (<20 vs. ≥20)	1.49	(1.06–2.08)	0.02

OR, odds ratio; CI, confidence interval.

**Table 4:** Multivariate logistic regression with dependent variable evolution to intensive care needs, adjusted for 25(OH)D status and all other variables.

Characteristics	OR Adjusted	(95% CI)	*p*-Value
Age ≥ 65 years	1.04	(0.77–1.4)	0.81
Cardiovascular	1.83	(1.33–2.52)	<0.001
Diabetes	1.22	(0.9–1.64)	0.189
Obesity	1.77	(1.33–2.35)	<0.001
Pulmonary diseases	1.55	(1.08–2.2)	0.015
Renal diseases	1.92	(1.2–2.98)	0.005
Hepatic diseases	1.09	(0.6–1.87)	0.762
Rheumatic diseases	0.69	(0.26–1.49)	0.391
Neurological diseases	1.91	(1.33–2.71)	<0.001
Cancer	1.6	(1.01–2.47)	0.037
Vaccine Doses (1 vs. 0)	0.7	(0.26–1.54)	0.417
Vaccine Doses (2 vs. 0)	0.59	(0.38–0.88)	0.013
Vaccine Doses (3 vs. 0)	0.13	(0.02–0.4)	0.004
Vitamin D (ng/mL) (<20 vs. ≥20)	1.18	(0.90–1.54)	0.235

OR, odds ratio; CI, confidence interval.

## Data Availability

Data available upon request due to restrictions, e.g., privacy or ethical concerns.

## Supplementary Materials

The following supporting information can be downloaded at https://www.mdpi.com/article/10.3390/nu15051227/s1, Table S1: Patients characteristics according to deficient, insufficient, and sufficient 25(OH)D; Table S2: Multivariate logistic regression with dependent variable severe/critical form of COVID-19 adjusted for 25(OH)D (deficient, insufficient, and sufficient) and all the other variables; Table S3: Multivariate logistic regression with dependent variable (evolution to death) adjusted for 25(OH)D (deficient, insufficient, and sufficient) and all the other variables; Table S4: Multivariate logistic regression with dependent variable evolution to intensive care need adjusted for 25(OH)D (deficient, insufficient, and sufficient) and all the other variables; Figure S1: Smoothing spline graphical representation of the relationship between 25(OH)D as a continuous variable and the log odds of severe/critical form in the multiple logistic regression model adjusted for Age ≥ 65 years, cardiovascular, diabetes, obesity, pulmonary diseases, renal diseases, hepatic diseases, rheumatic diseases, neurological diseases, cancer, vaccine doses; Figure S2: Smoothing spline graphical representation of the relationship between 25(OH)D as a continuous variable and the log odds of death in the multiple logistic regression model adjusted for Age ≥ 65 years, cardiovascular, diabetes, obesity, pulmonary diseases, renal diseases, hepatic diseases, rheumatic diseases, neurological diseases, cancer, vaccine doses.
