# Supplementary material for: 25 Hydroxyvitamin D Serum Concentration and COVID-19 Severity and Outcome—A Retrospective Survey in a Romanian Hospital
Source: Nutrients. 2023 Feb 28;15(5):1227. doi: 10.3390/nu15051227 (PMC10005256; doi:10.3390/nu15051227)
Supplement: Supplementary file 1 [file nutrients-15-01227-s001.zip › nutrients-2246034-supplementary.pdf]

Supplementary Table S1. Patients characteristics according to 25(OH)D deficiency, insufficiency and sufficiency

25(OH)D	<20 (n = 1194)	20–30 (n = 727)	≥30 (n = 421)	P
Age (years), median (IQR)	68 (52–78)	62 (47.5–72)	63 (50–71)	<0.001
Sex, n (%)				
Female	675 (56.53)	389 (53.51)	218 (51.78)	0.175
Male	519 (43.47)	338 (46.49)	203 (48.22)	
Cardiovascular, n (%)	769 (64.41)	396 (54.47)	247 (58.67)	<0.001
Diabetes, n (%)	283 (23.7)	144 (19.81)	82 (19.48)	0.062
Endocrine diseases, n (%)	63 (5.28)	66 (9.08)	47 (11.16)	< 0.001
Hepatic diseases, n (%)	66 (5.53)	32 (4.4)	29 (6.89)	0.195
Cancer, n (%)	115 (9.63)	54 (7.43)	28 (6.65)	0.086
Neurological diseases, n (%)	212 (17.76)	50 (6.88)	35 (8.31)	< 0.001
Obesity, n (%)	348 (29.15)	224 (30.81)	117 (27.79)	0.533
Pulmonary diseases, n (%)	168 (14.07)	73 (10.04)	46 (10.93)	0.022
Renal diseases, n (%)	83 (6.95)	42 (5.78)	20 (4.75)	0.234
Rheumatic diseases, n (%)	41 (3.43)	19 (2.61)	17 (4.04)	0.394
Intensive therapy, n (%)	157 (13.15)	78 (10.73)	36 (8.55)	0.028
Died, n (%)	118 (9.88)	34 (4.68)	27 (6.41)	< 0.001
Severe or critical, n (%)	560 (46.9)	279 (38.38)	153 (36.34)	< 0.001
Vaccinated, n (%)	254 (21.27)	149 (20.5)	92 (21.85)	0.851
Doses, n (%)				0.367
0:	940 (78.73)	578 (79.5)	329 (78.33)	
1:	31 (2.6)	24 (3.3)	6 (1.43)	
2:	171 (14.32)	91 (12.52)	68 (16.19)	
3:	52 (4.36)	34 (4.68)	17 (4.05)	

IQR, interquartile range; ICU, intensive care unit; *0/1, 0 means unvaccinated, 1 incomplete vaccination; **2, complete primary vaccination; ***3, booster dose.

Supplementary Table S2. Multivariate logistic regression with dependent variable severe/critical form of COVID-19 adjusted for 25(OH)D deficiency, insufficiency and sufficiency and all the other variables.

**Characteristics**	**OR adjusted (95% CI)**		**p**
Age ≥ 65 years	1.74	(1.42–2.13)	<0.001
Cardiovascular	1.86	(1.52–2.27)	<0.001

Diabetes	1.56	(1.26–1.94)	<0.001
Obesity	1.78	(1.46–2.18)	<0.001
Pulmonary diseases	1.32	(1.01–1.72)	0.043
Renal diseases	1.75	(1.21–2.53)	0.003
Hepatic diseases	1.11	(0.76–1.62)	0.581
Rheumatic diseases	1.58	(0.97–2.57)	0.066
Neurological diseases	1.84	(1.4–2.43)	<0.001
Cancer	1.3	(0.94–1.79)	0.108
Vaccine doses	0.7	(0.62–0.77)	<0.001
25(OH)D (<20 vs. 20-30)	1.18	(0.96–1.44)	0.107
25(OH)D (<20 vs. ≥30)	1.32	(1.03–1.69)	0.026

OR, odds ratio; CI, confidence interval.

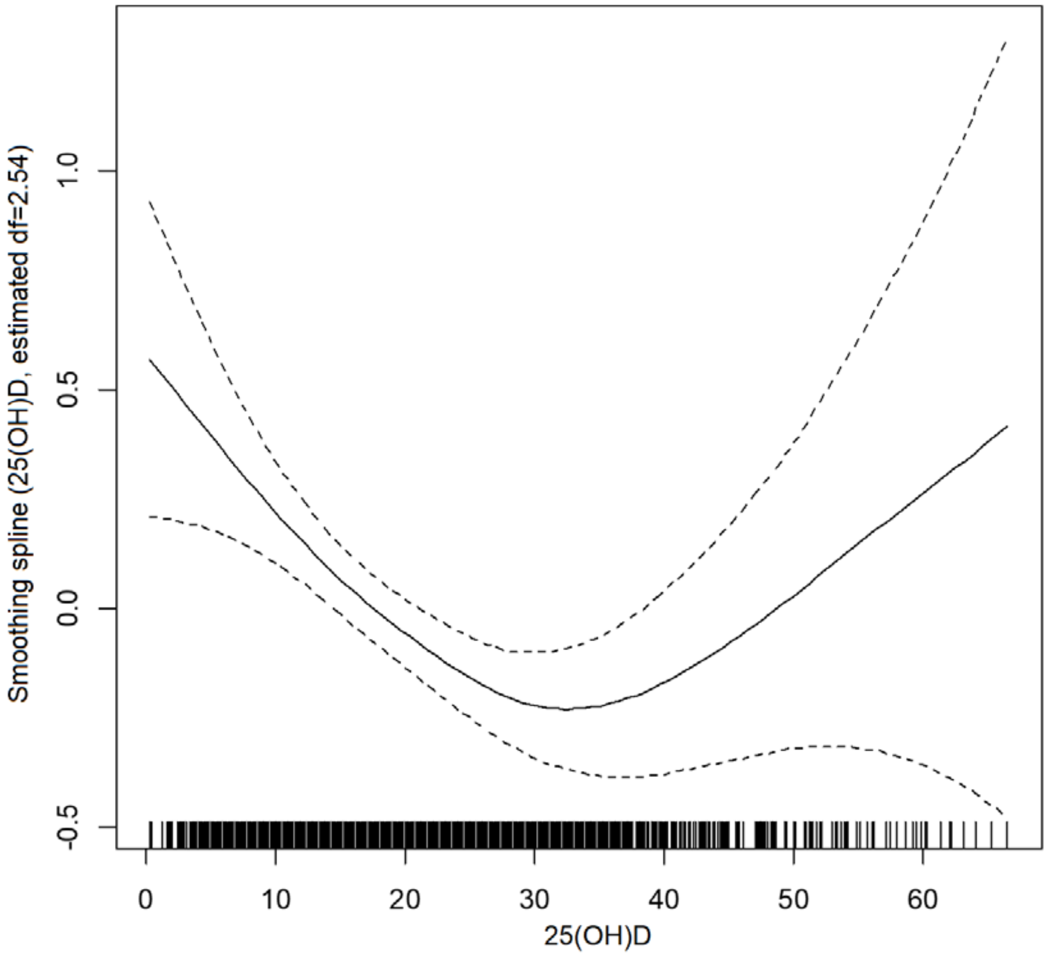

Supplementary Figure S1. Smoothing spline graphical representation of the relation between 25(OH)D as a continuous variable and the log odds of severe/critical form in the multiple logistic regression model adjusted for Age ≥ 65 years, cardiovascular, diabetes, obesity, pulmonary diseases, renal diseases, hepatic diseases, rheumatic diseases, neurological diseases, cancer, vaccine doses. The continuous line represents the smoothing spline, and the

dashed lines represent its confidence intervals. On the horizontal line there is a rugplot of the individual 25(OH)D values.

Suplementary Tabel S3. Multivariate logistic regression with dependent variable evolution to death adjusted for 25(OH)D deficiency, insufficiency and sufficiency and all the other variables.

**Characteristics**	**OR adjusted (95% CI)**		**p**
Age ≥ 65 years	2.91	(1.92–4.52)	<0.001
Cardio	2.36	(1.53–3.77)	<0.001
Diabetes	1.13	(0.79–1.6)	0.508
Obesity	1.41	(0.97–2.01)	0.066
Pulmonary diseases	1.16	(0.74–1.78)	0.492
Renal diseases	1.76	(1.03–2.87)	0.03
Hepatic diseases	1.01	(0.46–1.96)	0.985
Rheumatic disease	0.68	(0.2–1.71)	0.47
Neurological diseases	2.04	(1.38–2.99)	<0.001
Cancer	1.72	(1.01–2.8)	0.036
Vaccine doses	0.77	(0.62–0.94)	0.015
Vitamin D (<20 vs. 20-30)	1.69	(1.15–2.56)	0.01
Vitamin D (<20 vs. ≥30)	1.23	(0.79–1.96)	0.371

OR, odds ratio; CI, confidence interval.

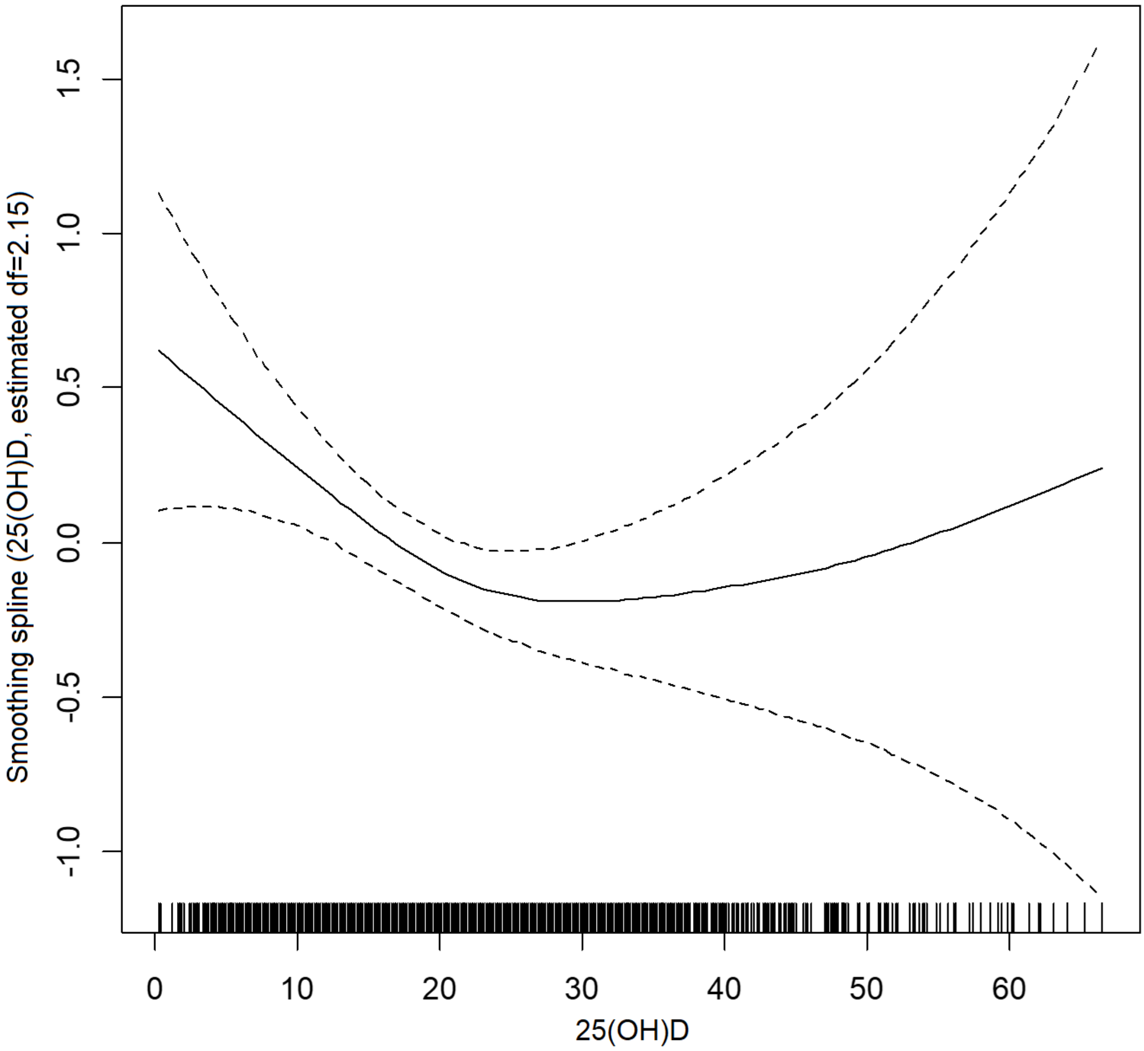

Supplementary Figure S2. Smoothing spline graphical representation of the relation between 25(OH)D as a continuous variable and the log odds of death in the multiple logistic regression model adjusted for Age ≥ 65 years, cardiovascular, diabetes, obesity, pulmonary diseases, renal diseases, hepatic diseases, rheumatic diseases, neurological diseases, cancer, vaccine doses. The continuous line represents the smoothing spline, and the dashed lines represent its confidence intervals. On the horizontal line there is a rugplot of the individual 25(OH)D values.

Suplementary Table S4. Multivariate logistic regression with dependent variable evolution to intensive care need adjusted for 25(OH)D deficiency, insufficiency and sufficiency) and all the other variables.

**Characteristics**	**OR adjusted**	**(95% CI)**	**p**
Age ≥ 65 years	1.04	(0.77–1.4)	0.823
Cardiovascular	1.84	(1.34–2.54)	<0.001
Diabetes	1.22	(0.9–1.64)	0.192

Obesity	1.78	(1.34–2.36)	<0.001
Pulmonary diseases	1.57	(1.09–2.23)	0.012
Renal diseases	1.93	(1.21–2.99)	0.004
Hepatic diseases	1.11	(0.61–1.9)	0.719
Rheumatic diseases	0.69	(0.26–1.5)	0.395
Neurological diseases	1.9	(1.32–2.7)	<0.001
Cancer	1.58	(1–2.43)	0.043
Vaccin doses	0.69	(0.57–0.82)	<0.001
Vitamin D (<20 vs. 20-30)	1.08	(0.80–1.45)	0.646
Vitamin D (<20 vs. ≥30)	1.39	(0.95–2.08)	0.101

OR, odds ratio; CI, confidence interval.
